# Analysis of Blood and Fecal Transcriptomes to Assess the Gastrointestinal Nematode Resistance in Naturally Exposed Katahdin Ewe

**DOI:** 10.1155/japr/3094932

**Published:** 2026-11-14

**Authors:** Chao Ke, Erin Shangraw, Men Su, Md. Imranuzzaman, Dipsana Kc, Prerana Karki, Tu Niya, Joel David Leal-Gutierrez, Thomas McFadden, Tumen Wuliji

**Affiliations:** ^1^ Division of Animal Science College of Agriculture Food and Natural Resources University of Missouri Columbia Missouri USA missouri.edu; ^2^ Department of Agriculture and Environmental Sciences Lincoln University Jefferson City Missouri USA lincolnuca.edu

## Abstract

Gastrointestinal nematodes (GIN) remain a major constraint to sheep production, a challenge intensified by widespread anthelmintic resistance. Host resistance to parasites is heritable, but scalable biomarkers for early selection are limited. The objective of this study was to compare phenotypic, hematological, and transcriptomic profiles of naturally exposed Katahdin ewes with contrasting fecal egg counts (FECs) to identify candidate genes associated with the observed phenotype. Seventy‐two adult ewes were screened on three occasions, and five ewes with low and five with high mean FEC were selected. Blood and fecal samples were collected in February and July 2025; group sizes were five low‐FEC and five high‐FEC ewes in February and five low‐FEC and four high‐FEC ewes in July. Low‐FEC ewes had lower FEC across subsequent sampling dates. Blood RNA sequencing (RNA‐seq) identified 19 differentially expressed genes (DEGs) in February and 13 in July, representing 29 unique genes. Three DEGs were shared between dates: two showed higher expression in low‐FEC ewes and one in high‐FEC ewes. No DEGs were detected in fecal RNA‐seq, although 8606 ovine genes were annotated. Comparison with an independent sheep expression atlas provided gastrointestinal reference annotations for a subset of these genes. Blood RNA‐seq identified candidate associations requiring independent validation, whereas fecal RNA‐seq demonstrated host‐transcript recovery without demonstrating an ability to distinguish the FEC groups in this cohort.

## 1. Introduction

Gastrointestinal nematode (GIN) infections are a major health threat to sheep worldwide ([1]), with haemonchosis caused by *Haemonchus contortus* representing one of the most economically important forms of parasitism in sheep and goats [2]. Reliance on anthelmintic drugs has led to widespread drug‐resistant parasite populations, making chemical control less effective [3, 4]. Consequently, breeding for parasite‐resistant sheep has emerged as a sustainable control strategy [5]. Resistance to GIN is a heritable trait; for example, in Creole goats, heritability estimates for fecal egg count (FEC), a common indicator of resistance to GIN infection, have ranged from approximately 0.2–0.4 depending on age and stage of production [6]. In this context, Katahdin hair sheep, a breed developed partly from the naturally parasite‐resistant St. Croix, have demonstrated genetic variation in GIN resistance, with moderate heritability for FEC (h^2^ ≈ 0.18–0.46 in lambs) [7]. Several phenotypic traits have been utilized as indicators of resistance and resilience to GINs, including FEC, hematocrit (HCT), packed cell volume (PCV), body condition score (BCS), and FAMACHA score [8, 9]. Nonetheless, the biological mechanisms behind host resistance remain incompletely understood, and few practical biomarkers are available to predict an individual’s parasite resilience early in life. Transcriptome profiling by RNA sequencing (RNA‐seq) can elucidate gene expression differences between resistant and susceptible animals, yielding insight into the immune genes and pathways associated with the host response to GIN infection [10]. Such differentially expressed genes (DEGs) may identify candidates for subsequent validation as biomarkers or for functional investigation. Several studies have investigated whole‐blood or blood‐cell transcriptomes [11]. Jacobs et al. [12] compared peripheral blood transcriptomes of susceptible Suffolk and resistant St. Croix sheep [12], in response to *H. contortus* infection. In another study, Bhuiyan et al. [13] reported blood transcriptomic profiling of *Yichang* White Goats that are classified as GIN susceptible and resistant [13]. To date, most transcriptomic studies of GIN infections in sheep have focused on tissues at the site of infection (abomasal mucosa, liver, and lymph nodes) [14–16]. These studies often require invasive sampling or slaughter, which is impractical for routine screening [17]. Therefore, there is a need for less invasive yet informative methods to assess mucosal immune responses to GIN in sheep.

Feces contain microbial material and shed host cells, including gastrointestinal (GI) epithelial cells and leukocytes. RNA recovered from these cells may provide information about mucosal processes [18]. In the context of colon in rats and humans, isolating and sequencing of mRNA from exfoliated cells has been validated, and human neonatal GI development was characterized [19, 20]. Thereafter, a noninvasive exfoliated cell RNA‐seq study in horses [21] and neonatal calves [17, 22–24] validated that the fecal exfoliome contained transcripts representing the diverse array of cell types found in the GI mucosa, supporting the use of the fecal RNA as a noninvasive tool to investigate GI transcriptome [21]. However, this approach has not been studied in parasitic infections or immunity in small ruminants. Our objectives were to compare blood and fecal transcriptomes of naturally exposed ewes with contrasting FEC phenotypes and to assess whether fecal RNA could recover host transcripts and detect expression differences between groups. We hypothesized that low‐FEC and high‐FEC ewes would differ in hematological traits and blood and fecal transcriptomic profiles.

## 2. Materials and Methods

### 2.1. Animal Selection

The research protocol describing all animal handling and sampling was approved by the Lincoln University Institutional Animal Care and Use Committee. A total of 72 adult ewes (3–5 years old), rotationally grazed on pasture at Freeman Farm (38°35 ^′^01 ^"^N 92°10 ^′^53 ^"^W) were screened for FEC on November 5, November 15, and December 20 in 2024. Fecal samples were collected directly from the rectum using lubricated, gloved hands. At the same time, live weight, body condition, and FAMACHA scores were recorded by experienced farm technicians. BCS was assessed using the sheep 1–5 scale [25]. Fecal samples from 72 ewes were analyzed for FEC using the modified quantitative McMaster flotation technique [26] at the Small Animals Research Facility laboratory, Lincoln University (Jefferson City, Missouri, United States). Briefly, 4 g of feces were homogenized in 56 mL of 40% sodium chloride solution using a mechanical blender. The homogenized mixture was filtered through two layers of cheesecloth; the filtered mixture was then aliquoted using a transfer pipette into two chambers of the McMaster slide and left on the bench for at least 5 min but no more than 15 min to ensure parasite eggs floated to the surface. Eggs within the grid area of both chambers were counted under the compound microscope at 10 × magnification. Finally, FEC was expressed as eggs per gram (EPG) of feces and calculated by multiplying the total number of eggs counted in both chambers by a dilution factor of 50 [26]. FEC represented total strongyle‐type EPG of feces and was not species specific. Larval culture and PCR‐based identification of parasite species were not performed in this study. The ewes had been maintained in the same flock and pasture for years (with age range 3–5) and had undergone annual FEC screening before this study. For each ewe, the arithmetic mean of FEC was calculated across November 5, November 15, and December 20, 2024. The 72 ewes were ranked by mean FEC, and the five ewes with the lowest values and the five with the highest values were selected as the low‐FEC and high‐FEC groups, respectively. FAMACHA score, BCS, live weight, HCT, and estimated breeding values were not used as criteria for selection or group assignment.

### 2.2. Sample Collection

Blood and fecal samples from the selected ewes were collected at two reproductive stages: during pregnancy on February 26, 2025, and during late lactation on July 1, 2025. Group sizes were five low‐FEC and five high‐FEC ewes in February and five low‐FEC and four high‐FEC ewes in July. Live weight and BCS were recorded for each animal before sampling. The blood samples were collected from the jugular vein into Tempus™ Blood RNA Tubes (Thermo Fisher, Waltham, Massachusetts) and BD Vacutainer Plastic EDTA Tubes (Thermo Fisher, Waltham, Massachusetts). For each ewe, one scoop of fecal sample was collected from the rectum and immediately placed into DNA/RNA Shield Fecal Collection Tubes (ZYMO research, Irvine, California) based on the manufacturer’s instructions. Both blood (samples in Tempus™ Blood RNA Tubes) and fecal samples were then transported to the laboratory and stored at −80°C pending RNA extraction. Blood samples collected into EDTA tubes were analyzed using a Hemavet 950 automated veterinary hematology analyzer (Drew Scientific), according to the manufacturer’s instructions.

### 2.3. RNA Extraction

For blood, total RNA was obtained using a MagMax RNA isolation kit. Tempus‐stabilized samples stored in the frozen state were thawed on ice for 10–30 min. The full contents of each tube were transferred to a clean, labeled 50‐mL conical tube, followed by the addition of 3 mL of Tempus 1× PBS (MagMAX for Stabilized Blood Tubes RNA Isolation Kit) to obtain a final volume of 12 mL. Samples were centrifuged at 5000 × g for 15 min at 4°C to pellet crude RNA, after which the supernatant was carefully removed, and 1 mL of TRIzol reagent (Thermo Fisher Scientific, Massachusetts, United States) and 200 *μ*L of chloroform (Thermo Fisher Scientific, Massachusetts, United States) were then added and mixed well, and RNA was washed and collected using Qiagen Rneasy Mini Kit based on the manufacturer’s instructions. Total RNA was extracted from feces using Quick‐RNA Fecal/Soil Microbe Microprep Kit (ZYMO Research). The total RNA concentration was measured using a NanoDrop One/OneC Microvolume UV‐Vis Spectrophotometer (Thermo Fisher Scientific, Massachusetts, United States), and RNA integrity was assessed using a 5200 Fragment Analyzer (Agilent), with RNA quality number (RQN) determined using ProSize software Version 4.0.2.7 (MU Genomics Technology Core, Columbia, Missouri, United States). The extracted total RNA was stored at −80°C until use. The cDNA fragment analysis was conducted using a Takara ultralow input SMART‐Seq kit. For blood samples, 5 ng of isolated RNA was used, and cDNA was amplified by 11 cycles of PCR. For fecal samples, 50 ng of RNA was used, and cDNA was amplified by 13 PCR cycles.

### 2.4. Library Preparation and Sequencing

Libraries were constructed following the manufacturer’s protocol with reagents supplied in SMART‐Seq mRNA LP (with UMIs) (Takara Bio, cat. No. 634762). An input of 5–50 ng of total RNA was used to synthesize double‐stranded cDNA. Priming was performed with the SMART‐Seq mRNA Oligo(dT) primer and the SMART‐Seq mRNA UMI TSO for template switching at the 5 ^′^ end of the transcript. First strand synthesis was performed with SMARTScribe reverse transcriptase and cDNA synthesis was performed by long‐distance PCR with SeqAmp DNA polymerase. Amplified cDNA was purified by AxyPrep Mag PCR Clean‐Up magnetic beads. The cDNA samples were enzymatically fragmented, adaptors ligated and libraries amplified according to the protocol. The barcodes supplied in the kit were incorporated in the final amplification cycles. Purified libraries were quantified using the Qubit assay (Thermo Fisher Scientific, Massachusetts, United States) and library fragment size was confirmed by analysis on a 5200 Fragment Analyzer (Agilent) using the High Sensitivity DNA assay reagents (Agilent, Santa Clara, California, United States). Libraries were diluted and sequenced according to the standard sequencing protocol on a NovaSeq X (Illumina; 100‐bp paired‐end reads). A sequencing depth of fifty million reads per sample was targeted.

### 2.5. Quality Control and Mapping to the Reference Genome

Adapter and quality trimming were performed using fastp. Poly‐G tail trimming was enabled; bases were filtered at a minimum quality threshold of Q20, and reads shorter than 25 bp were discarded. Trimmed paired reads and unpaired reads were retained. Quality control of trimmed FASTQ files was performed using FastQC. Reads were aligned to the *Ovis aries* reference genome assembly NWAFU_Friesian_1.0 (GCA_018804185.1) obtained from Ensembl, together with the corresponding Ensembl gene annotation (GTF) file. A STAR genome index was generated from the concatenated genome FASTA file and the Ensembl GTF annotation file [27]. Trimmed paired reads were aligned to the STAR genome index, and gene‐level counts were generated using featureCounts. Differential expression was analyzed from gene‐level counts using the Bioconductor package DESeq2 [28] in R 4.5.0. [29]. Differential expressions were evaluated separately for each sampling date. Log_2_ fold change was defined as high‐FEC relative to low‐FEC, where positive values indicate higher expression in high‐FEC ewes and negative values indicate higher expression in low‐FEC ewes. DEGs were defined by a Benjamini–Hochberg‐adjusted *p* value ≤ 0.05 and |log_2_ fold change| > 1.

### 2.6. GI Reference Categorization of Genes Detected in Fecal RNA‐seq

Reference expression profiles were obtained from Expression Atlas [30], using the sheep dataset E‐MTAB‐3838 described in the associated publication [31]. Two reference files were downloaded, including the transcripts per million (TPM) matrix (E‐MTAB‐3838‐tpms.tsv) and the corresponding sample metadata file (E‐MTAB‐3838.sdrf.txt). The atlas TPM columns were mapped to biological tissues using the accompanying SDRF metadata. Atlas tissues were then collapsed into GI compartments relevant to ruminant biology. Tissue labels matching rumen, reticulum, omasum, and abomasum were assigned individually. Labels matching duodenum, jejunum, ileum, and small intestine were grouped as small intestine. Samples labeled as cecum, colon, rectum, or large intestine were grouped as large intestine. Annotated genes from fecal RNA samples in this study were matched with atlas TPM files and gene categorization was summarized. A detection threshold of mean TPM ≥ 1 was applied to define expression presence within a GI compartment, and the primary and secondary GI reference compartments were assigned according to the highest and second highest mean TPM values, respectively. These assignments describe expression patterns in independent reference tissues and do not identify the tissue of origin of transcripts recovered from the fecal samples.

### 2.7. Functional Enrichment Analysis

Functional enrichment analysis was performed separately for the February and July blood transcriptomes using clusterProfiler v4.16.0 in R v4.5.0 [32]. Ensembl gene identifiers were annotated using the NWAFU_Friesian_1.0 assembly and Ensembl release 114 GTF file, then linked to sheep Entrez Gene identifiers and Gene Ontology (GO) annotations using AnnotationHub resource AH120062. GO biological process, molecular function, and cellular component categories and sheep Kyoto Encyclopedia of Genes and Genomes (KEGG; organism code oas) pathways were evaluated. Reactome analysis used human pathways from release 97 after mapping sheep genes to unambiguous human orthologs.

Over‐representation analysis (ORA) used DEGs meeting a false discovery rate (FDR)‐adjusted *p* value ≤ 0.05 and |log_2_ fold change| > 1, with the background restricted to genes tested in the corresponding DESeq2 comparison and annotated in the relevant database. Preranked gene set enrichment analysis (GSEA) used genes with nonmissing DESeq2 Wald statistics, ranked by the signed statistic without a DEG significance filter [33]. Gene sets containing 10–500 mapped genes were evaluated. *p* values were adjusted using the Benjamini–Hochberg procedure, with adjusted *p* ≤ 0.05 considered significant.

### 2.8. Statistical Analyses

Hematological traits were analyzed using linear mixed‐effects models with selection group, sampling time, and their interaction as fixed effects and ewe ID as a random intercept. Post hoc group comparisons within each time point were performed using model‐based contrasts with two‐sided Wald tests. Data are presented as standard error of the mean (SEM). Statistical significance was assessed at *p* < 0.05; *p* < 0.01 is indicated separately in the figures.

## 3. Results

### 3.1. Classification of Low‐FEC and High‐FEC Ewes

From 72 pasture‐grazed Katahdin ewes, five animals with low and five with high mean FEC across three screening dates (November 5, November 15, and December 20, 2024) were selected. Group sizes were five low‐FEC and five high‐FEC ewes in February and five low‐FEC and four high‐FEC ewes in July. Low‐FEC ewes exhibited lower FEC than high‐FEC ewes across subsequent sampling dates (*p* = 0.003; Figure 1). Across the study period, mean FEC was 140 ± 63 EPG in low‐FEC ewes and 2174 ± 246 EPG in high‐FEC ewes (*p* < 0.01, Table 1). Mean live weight was 52.64 ± 1.56 kg in low‐FEC ewes and 45.00 ± 1.44 kg in high‐FEC ewes (*p* = 0.12). FAMACHA scores and BCS did not differ significantly between groups (Table 1).

**Figure 1:**
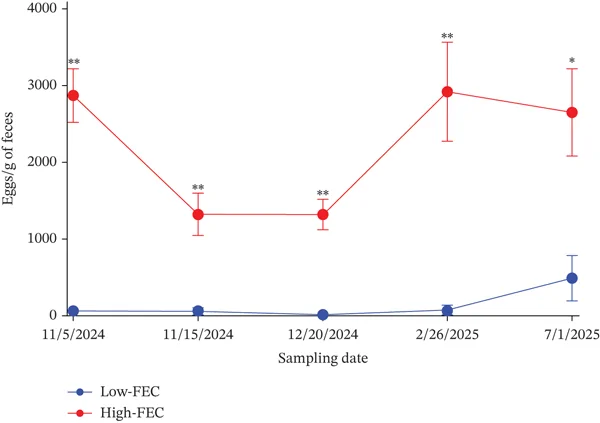
Longitudinal FEC in low‐FEC and high‐FEC Katahdin ewes. Mean eggs per gram (EPG) of feces are shown across five sampling dates. Group sizes were five low‐FEC and five high‐FEC ewes through February 2025 and five low‐FEC and four high‐FEC ewes in July 2025. Points represent group means, and error bars denote the standard error of the mean (SEM). Asterisks indicate statistically significant differences between the low‐FEC and high‐FEC groups at the corresponding sampling dates ( ^∗^ *p* < 0.05;  ^∗∗^ *p* < 0.01).

**Table 1:** Fecal egg counts (FEC), FAMACHA scores, live weight and body condition scores (BCS) in low‐FEC and high‐FEC Katahdin ewes. Values summarize measurements across the study period; group sizes were five low‐FEC and five high‐FEC ewes through February 2025 and five low‐FEC and four high‐FEC ewes in July 2025.

Parameters	Low‐FEC group	High‐FEC group
FEC (eggs/gram)	140 ± 63^∗∗^	2174 ± 246
Live weight (kg)	52.64 ± 1.56	45.00 ± 1.44
FAMACHA	1.55 ± 0.14	1.42 ± 0.11
BCS	2.68 ± 0.22	2.42 ± 0.14

*Note:* Values are presented as standard error of the mean (SEM).

*p* < 0.05.

^∗∗^ *p* < 0.01.

### 3.2. Hematology Profiles

Longitudinal changes in leukocyte and erythrocyte numbers differed between low‐FEC and high‐FEC ewes across the four sampling time points, with the final sampling conducted during the postlambing period (Figure 2). Low‐FEC ewes consistently exhibited higher white blood cell counts than high‐FEC ewes, with significant (*p* = 0.013) group differences observed in December 2024 and February 2025 (Figure 2a), indicating differences in circulating leukocyte counts. HCT was also significantly higher in Low‐FEC ewes at multiple time points (Figure 2b), consistent with greater resilience to GIN. Eosinophil counts were higher in Low‐FEC ewes across the study, with the largest numerical divergence observed in February 2025 (Figure 2c), although between‐group differences did not reach statistical significance. Lymphocyte counts followed a similar temporal pattern and were significantly higher in low‐FEC ewes at the December 2024 sampling (*p* = 0.032) (Figure 2d). Following lambing, lymphocyte counts declined in both groups, with a greater relative decrease in low‐FEC ewes, potentially reflecting periparturient immunological modulation.

**Figure 2:**
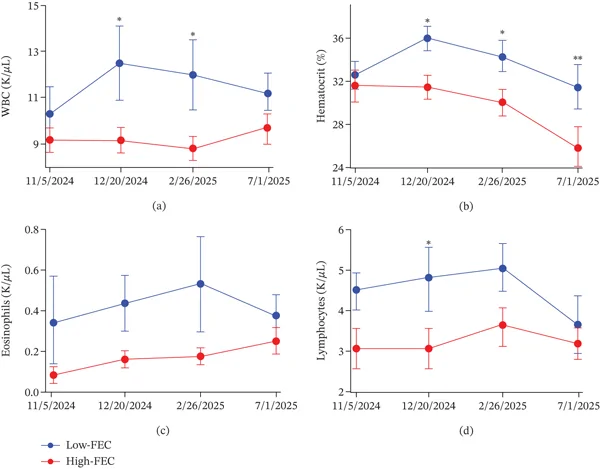
Mean (±standard error of the mean, SEM) values of (a) white blood cell (WBC) count, (b) hematocrit (HCT), (c) eosinophils, and (d) lymphocytes in low‐FEC (blue) and high‐FEC (red) ewes at four sampling dates. Group sizes were five low‐FEC and five high‐FEC ewes through February 2025 and five low‐FEC and four high‐FEC ewes in July 2025. The final sampling was conducted during late lactation. Resistant and susceptible labels in the artwork denote the operational low‐FEC and high‐FEC groups, respectively. Asterisks represent significant differences between groups at individual time points ( ^∗^ *p* < 0.05;  ^∗∗^ *p* < 0.01).

### 3.3. Sequencing Performance and Host Mapping

Blood RNA had RQN values of 10.0 in February and 9.8–10.0 in July. The nine July fecal RNA preparations had a mean RQN of 8.46 (range, 6.5–9.2). Sequencing generated 10 blood and 10 fecal libraries in February and 9 blood and 9 fecal libraries in July. Across total of 38 samples, the mean sequencing output was 54,132,665 read pairs per library (SD = 6,749,338). On average, 85.70% of blood RNA‐seq reads mapped to the ovine reference genome NWAFU_Friesian_1.0 (GCA_018804185.1; SD = 0.90 percentage points). Fecal RNA‐seq samples had a mean host‐mapping rate of 1.11% (SD = 1.50 percentage points, range, 0.32%–5.35%; median, 0.72%) (Table S2). The alignment performance observed in blood samples is comparable with previously reported ovine RNA‐Seq studies using blood, which typically report mapping efficiencies in the range of 78%–89.91% [13, 34].

### 3.4. Differential Gene Expression

Blood RNA‐seq identified 19 DEGs in February 2025 (five low‐FEC and five high‐FEC ewes): 14 showed higher expression in low‐FEC ewes and five in high‐FEC ewes (Benjamini–Hochberg‐adjusted *p* ≤ 0.05; |log_2_ fold change| > 1) (Figure 3a). In July 2025 (five low‐FEC and four high‐FEC ewes), 13 genes were differentially expressed, with nine genes showing higher expression in low‐FEC ewes and four in high‐FEC ewes (Figure 3b). Genes exhibiting higher expression in low‐FEC ewes included 5 ^′^‐aminolevulinate synthase 2 (*ALAS2*), threonyl‐tRNA synthetase 3 (*TARS3*), protein tyrosine phosphatase receptor type Q (*PTPRQ*), ATP synthase inhibitory factor subunit 1 (*ATP5IF1*), adhesion G protein‐coupled receptor E3 (*ADGRE3*), haloacid dehalogenase like hydrolase domain containing 2 (*HDHD2*), complement C3a receptor 1 (C3AR1), and chromosome 6 open reading frame 52 (*C6orf52*), whereas transmembrane protein 158 (*TMEM158*), bone marrow stromal cell antigen 2B (*BST2B*), transcription factor EC (*TFEC*), adenylate kinase 5 (*AK5*)*, synuclein alpha* (*SNCA*), and ribosomal protein S3A (*RPS3A*) showed higher expression in high‐FEC ewes. Three genes were differentially expressed at both sampling dates: ENSOARG00040049122 and ENSOARG00040049125 showed higher expression in low‐FEC ewes, whereas ENSOARG00040012475 showed higher expression in high‐FEC ewes. *RPS3A* showed higher expression in high‐FEC ewes in July (log_2_ fold change = 3.682; Benjamini–Hochberg‐adjusted *p* = 0.049492)*. PTPRQ* showed higher expression in low‐FEC ewes in February; the reported *p* value in July was 0.05. No DEGs were detected in fecal samples at either sampling time. The small cohort and host‐mapping rate may have limited the sensitivity of the fecal differential expression analysis.

**Figure 3:**
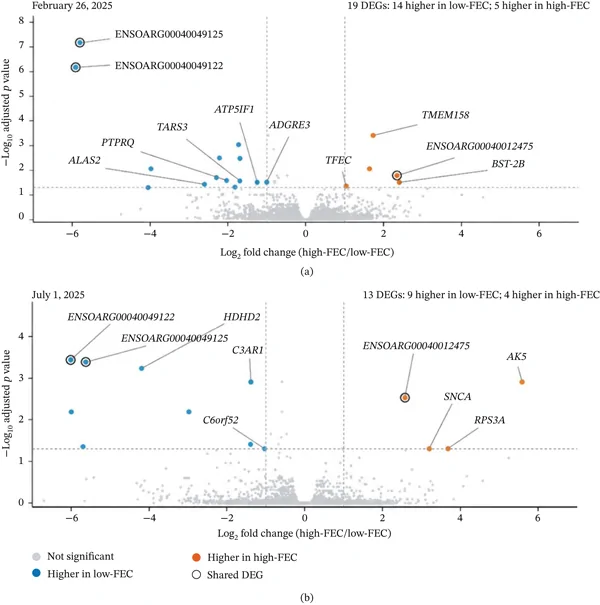
Blood transcriptomic differences between low‐FEC and high‐FEC of Katahdin ewes. Volcano plots show (a) February 26, 2025 (five low‐FEC and five high‐FEC ewes) and (b) July 1, 2025 (five low‐FEC and four high‐FEC ewes). Each point represents a gene with a finite log_2_ fold change and adjusted *p* value. Log_2_ fold change is defined as high‐FEC relative to low‐FEC: positive values indicate higher expression in high‐FEC ewes and negative values indicate higher expression in low‐FEC ewes. R and S in the artwork denote the operational low‐FEC and high‐FEC groups, respectively. Blue and orange points meet both DEG criteria (Benjamini–Hochberg‐adjusted *p* ≤ 0.05 and |log_2_ fold change| > 1); gray points do not. Dashed lines mark the thresholds. Black rings identify the three DEGs shared between dates, labeled by Ensembl identifier. Named DEGs are labeled by their supplied gene annotations. Panel headings report total and direction‐specific DEG counts. The *x*‐axis range is identical across panels; *y*‐axis ranges differ.

### 3.5. GI Reference Annotation of Fecal Host Transcripts

Among the 8606 ovine genes annotated in fecal RNA‐seq, genes with matching GI reference expression profiles were categorized using the sheep expression atlas (Table S1). The matched records were summarized into nonexclusive functional categories, including immune and inflammatory signaling, epithelial and mucosal functions, energy metabolism, lipid and carbohydrate metabolism, intracellular signaling, cellular maintenance, adhesion, and transport. Immune‐associated genes are summarized in Table 2, with their primary and secondary reference compartments and atlas‐derived TPM ranges. These annotations describe reference‐tissue expression patterns rather than tissue‐specific expression measured in the study animals.

**Table 2:** Immune‐associated genes detected in fecal RNA‐seq and their gastrointestinal reference‐atlas expression annotations.

Gene symbol	Primary reference compartment	Secondary reference compartment	Immune‐associated function	Primary atlas TPM range	Secondary atlas TPM range
TLR6	Rumen	Abomasum	Pattern‐recognition signaling	2–7	3–8
STAT1	Rumen	Small intestine	Interferon signaling	12–70	21–21
CXCL8	Rumen	None	Chemokine signaling	0–13	NA
CD247	Abomasum	Rumen	T‐cell receptor signaling	7–88	3–22
CD3D	Abomasum	Rumen	T‐cell receptor complex	0.7–49	0.1–24
LYZ	Abomasum	Rumen	Innate effector function	1–150	0.3–37
CD3E	Abomasum	Small intestine	T‐cell receptor complex	0.8–70	10–10
FCGR3A	Abomasum	Small intestine	Myeloid‐cell receptor signaling	4–239	45–45
ITGAL	Abomasum	Small intestine	Leukocyte adhesion	2–30	5–5
ITGB2	Abomasum	Small intestine	Leukocyte adhesion	2–48	10–10
LYN	Abomasum	Small intestine	Myeloid and B‐cell signaling	2–46	15–15
MRC1	Abomasum	Small intestine	Myeloid‐cell recognition	0.9–111	27–27
SERPINB1	Abomasum	Small intestine	Myeloid‐cell effector function	3–41	12–12
CD52	Abomasum	Large intestine	Lymphocyte signaling	3–211	0–240
IDO1	Abomasum	Large intestine	Immune‐metabolic regulation	2–7	2–6
LCK	Abomasum	Large intestine	T‐cell receptor signaling	0.2–31	0.4–24
RHOH	Abomasum	Large intestine	Leukocyte signaling	0.2–59	0–57
IL1B	Abomasum	None	Inflammatory cytokine signaling	0.2–2	NA
TNF	Abomasum	None	Inflammatory cytokine signaling	0.7–12	NA
ITGB6	Small intestine	Rumen	Mucosal cell adhesion	13–13	0.1–17
TAPBP	Small intestine	Rumen	Antigen processing and presentation	21–21	5–25
B2M	Small intestine	Abomasum	Antigen presentation and immune surveillance	3397–3397	364–6293
CXCL10	Small intestine	Abomasum	Chemokine signaling	95–95	7–94
IL15	Small intestine	Abomasum	Cytokine signaling	9–9	0.9–15
IL1A	Small intestine	Abomasum	Inflammatory cytokine signaling	4–4	2–4
IRAK4	Small intestine	Abomasum	Innate receptor signaling	12–12	4–15
IRF2	Small intestine	Abomasum	Interferon regulation	23–23	11–36
ITGAM	Small intestine	Abomasum	Myeloid‐cell adhesion	7–7	1–10
ITGAV	Small intestine	Abomasum	Cell adhesion and leukocyte biology	34–34	27–32
ITGB1	Small intestine	Abomasum	Cell adhesion and leukocyte biology	133–133	45–189
NFKBIA	Small intestine	Abomasum	NF‐*κ*B regulation	16–16	5–25
PSMB8	Small intestine	Abomasum	Antigen processing	8–8	0.8–17
TLR2	Small intestine	Abomasum	Pattern‐recognition signaling	16–16	1–17
TLR4	Small intestine	Abomasum	Pattern‐recognition signaling	12–12	1–23
ISG15	Small intestine	Large intestine	Interferon‐stimulated response	75–75	0–75
MX1	Small intestine	Large intestine	Interferon‐stimulated response	101–101	2–101
MX2	Small intestine	Large intestine	Interferon‐stimulated response	26–26	0.4–26
MYD88	Small intestine	Large intestine	Innate receptor signaling	12–12	0.8–14
IL2RA	Small intestine	None	T‐cell activation	1–1	NA
CD53	Large intestine	Abomasum	Leukocyte membrane signaling	0.4–81	1–27
CXCL1	None	None	Chemokine signaling	NA	NA

*Note:* Primary and secondary reference compartments were assigned by ranking mean expression across gastrointestinal compartments in the reference atlas. Transcripts per million (TPM) ranges represent values in the corresponding reference‐atlas tissue samples, rather than measurements in gastrointestinal tissues of the study ewes. NA denotes an unavailable value; none denotes an unassigned reference compartment.

### 3.6. Functional Enrichment Analysis of Blood Transcriptomes

February GSEA identified significant GO biological process categories related to biotic‐stimulus responses, host defense, and T‐cell receptor signaling (Figure 4a). KEGG enrichment included antigen processing and presentation, Toll‐like receptor signaling, protein processing in the endoplasmic reticulum, lysosome biogenesis, and oxidative phosphorylation (Figure 4b). Reactome analysis identified interferon signaling, the ER–phagosome pathway, antigen processing and cross‐presentation, and Toll‐like receptor signaling (Figure 4c). February GO molecular‐function results are shown in Figure S1a; no February GO cellular‐component category met the adjusted *p* ≤ 0.05 threshold. July GSEA identified defense‐response and innate–immune‐response categories together with translation and ribonucleoprotein complex biogenesis (Figure 5a). Ribosome and oxidative phosphorylation were significantly enriched in KEGG (Figure 5b), whereas Reactome enrichment included translation initiation, elongation, termination, and ribosome‐associated quality control (Figure 5c). July GO molecular‐function and cellular‐component results also included structural constituent of ribosome and multiple ribosome‐related terms (Figure S1b–c). ORA yielded fewer significant categories. Significant February KEGG and Reactome terms were each represented by one DEG (Figure S2a–b). In July, one KEGG term was significant and contained two DEGs, whereas no Reactome term met the significance threshold (Figure S2c–d).

**Figure 4:**
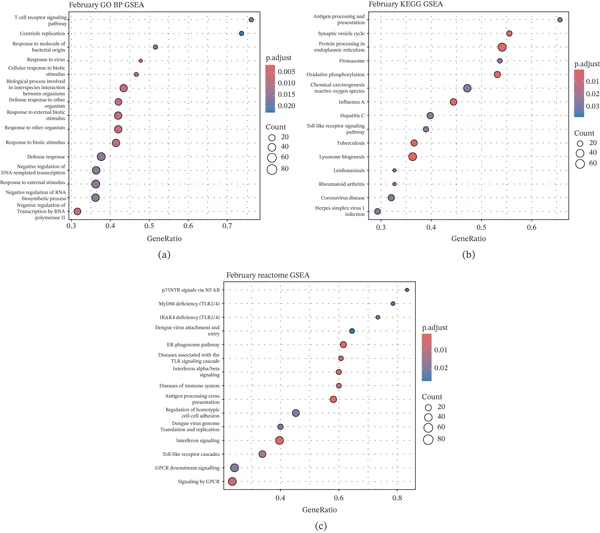
Functional enrichment of blood transcripts at the February sampling (five low‐FEC and five high‐FEC ewes). (a) Gene Ontology (GO) biological process, (b) Kyoto Encyclopedia of Genes and Genomes (KEGG), and (c) Reactome gene set enrichment analysis (GSEA) using human orthologs of sheep genes. Genes were ranked by the signed DESeq2 Wald statistic. Point size indicates the number of core‐enrichment genes, GeneRatio indicates their proportion within the corresponding gene set, and color denotes the Benjamini–Hochberg‐adjusted *p* value. All displayed categories and pathways met adjusted *p* ≤ 0.05; disease‐associated labels refer to database annotations.

**Figure 5:**
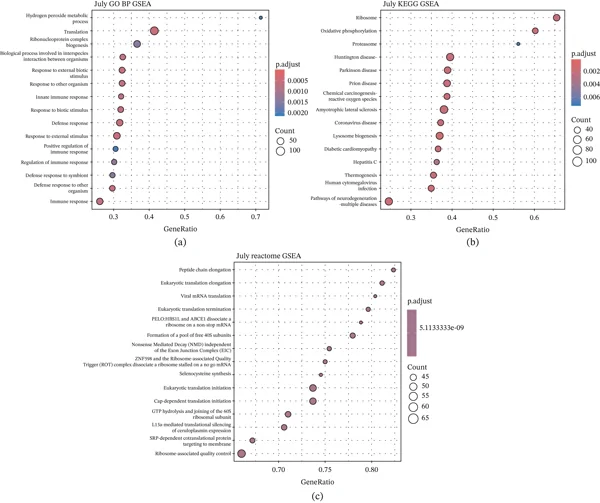
Functional enrichment of blood transcripts at the July sampling. (five low‐FEC and four high‐FEC ewes). (a) Gene Ontology (GO) biological process, (b) Kyoto Encyclopedia of Genes and Genomes (KEGG), and (c) Reactome gene set enrichment analysis (GSEA) using human orthologs of sheep genes. Genes were ranked by the signed DESeq2 Wald statistic. Point size indicates the number of core‐enrichment genes, GeneRatio indicates their proportion within the corresponding gene set, and color denotes the Benjamini–Hochberg‐adjusted *p* value. All displayed categories and pathways met adjusted *p* ≤ 0.05; disease‐associated labels refer to database annotations.

## 4. Discussion

### 4.1. Hematological Profiles

This study compared blood and fecal transcriptomic profiles of naturally exposed Katahdin ewes with contrasting FEC phenotypes. Low‐FEC ewes had significantly lower FECs and maintained higher HCT compared with high‐FEC ewes, consistent with previous reports that resistant animals avoid anemia in highly parasitized paddock and negative correlation between HCT and FEC [35, 36]. In addition to an increase in the numbers of WBC, nematode infection is also associated with eosinophil responses [37]. Eosinophils originate from hematopoietic stem cells in the bone marrow [38], and their development and survival are mediated by the Th2 immune responses, which are crucial for GIN resistance [39]. Accordingly, eosinophils have been recognized as important effector cells in the protective immune response against GIN infection in sheep [40, 41]. Peripheral blood eosinophil counts decreased in low‐FEC ewes in July 2025. Seasonal exposure and physiological changes associated with lactation are possible explanations for the observed patterns, but individual worm burdens and eosinophil recruitment to infected tissues were not measured. The study of Pacheco et al. [42] indicated that Th1 and Th2 immune traits derived from circulating lymphocytes contribute to resistance to both coccidian and nematode parasites [42], supported by a higher circulating lymphocyte count in low‐FEC ewes in this study.

### 4.2. Immune‐related DEGs

Several DEGs identified in the present study were linked to innate immune signaling, leukocyte recruitment, and immunometabolism. *C3AR1* and *ADGRE3* were highly expressed in the low‐FEC group. A previous genomic study identified a selection signal involving C3AR1 in sheep exposed to *H. contortus* [43], supporting its prioritization for further investigation. *C3AR1* is a G protein‐coupled receptor (GPCR) that mediates neutrophil or monocytes‐driven inflammation when bound by C3a, suggesting a role of mediator in inflammation [44, 45]; however, its contribution to ovine GIN resistance has not been demonstrated.

*ADGRE3* transcript abundance was higher in blood from low‐FEC ewes in the February comparison. This observation identifies *ADGRE3* as a candidate associated with the FEC phenotype, whereas its contribution to ovine GIN resistance remains unresolved. Variation in blood cell composition is a possible alternative explanation that requires further investigation.

ATP5IF1 and ALAS2 were also more highly expressed in the low‐FEC group. ATP5IF1 encodes ATP synthase inhibitory factor 1. Its association with mitochondrial metabolism has been reported in cancer cells, and mouse studies have implicated IF1 in the metabolic reprogramming of activated T cells [46] [47]. Likewise, *5-ALAS2* is an erythroid‐specific, rate‐limiting enzyme for hemoglobin production and red blood cell maturation [48, 49]. A recent study in fish macrophages shows that *ALAS2*‐driven heme accumulation promotes proinflammatory cytokine output [50]. Because the samples were derived from whole blood, higher ALAS2 transcript abundance may reflect differences in erythroid‐cell or reticulocyte contribution or altered erythropoietic activity. *RPS3A* showed higher transcript abundance in blood from high‐FEC ewes in July. This association does not establish a specific role in inflammation or GIN resistance. Its relationship with the FEC phenotype requires validation in an independent cohort. *BST2B* also showed higher transcript abundance in high‐FEC ewes; its contribution to the observed phenotype remains unresolved. Overall, these findings generate testable hypotheses involving complement and leukocyte biology, cellular metabolism, erythropoiesis, and protein synthesis, but they do not establish gene‐specific mechanisms of GIN resistance. Independent cohorts and targeted expression assays are required to determine whether these transcripts represent reproducible biological associations or useful biomarkers.

### 4.3. Host Transcript Recovery in Fecal RNA‐seq

No DEGS were detected in the fecal transcriptomic dataset. Nevertheless, 8606 annotated ovine genes were recovered, demonstrating that host transcripts can be detected in fecal material. These transcripts included immune‐associated genes such as *IL1B* and *TNF*. Both genes were assigned to the abomasum as their primary GI reference compartment on the basis of atlas expression profiles [51]. These assignments provide no direct evidence that the transcripts originated from the abomasum of the sampled ewes. The mean host‐mapping rate was 1.11% (SD = 1.50 percentage points), indicating limited recovery of ovine reads relative to the total sequenced reads. Microbial, plant‐derived, and parasitic RNA, together with RNA degradation, may contribute to the low proportion of reads mapping to the ovine genome. The composition of unmapped reads was not characterized in this study, and the relative contributions of these factors remain unresolved. Thus, the fecal data demonstrate recovery of ovine transcripts but provide no evidence of differential expression associated with the FEC phenotype.

### 4.4. Functional Enrichment of Blood Transcriptomes

At the February sampling, enrichment of gene sets associated with responses to biotic stimuli, interferon signaling, Toll‐like receptor signaling, antigen processing and presentation, and related immune processes indicated coordinated differences in systemic immune‐associated transcription between the two phenotypic groups. Similar classes of pathways have been reported in previous Ovine GIN transcriptomic studies. Chitneedi et al. identified enrichment of cytokine‐mediated immune‐response pathways in sheep differing in resistance to *Teladorsagia circumcincta*, whereas Zhang et al. reported resistance‐associated changes involving complement, leukocyte migration, coagulation, and tissue‐repair pathways during *H. contortus* infection [14, 52]. The concordance with previous ovine GIN transcriptomic studies supports an association between these immune‐related pathways and the host response to nematode infection; however, the present enrichment patterns cannot distinguish pathways contributing directly to resistance from those reflecting ongoing or delayed immune stimulation.

The functional profile differed substantially during the July sampling. High‐FEC ewes showed prominent enrichment of ribosomal, translational, oxidative‐phosphorylation, and related protein‐processing pathways, whereas several defense‐ and innate–immune‐response gene sets were enriched toward low‐FEC ewes. Protein‐synthesis and inflammatory networks have also been identified in ovine lymphoid transcriptomes associated with nematode resistance, although the direction and magnitude of these responses vary among tissues, breeds, parasite species, and sampling times. McRae et al. demonstrated that resistance‐associated inflammatory and leukocyte‐recruitment responses differed according to the stage of *T. circumcincta* infection, emphasizing that temporal context can substantially influence transcriptomic comparisons between resistant and susceptible sheep [10]. The contrasting enrichment directions of several immune‐related gene sets between February and July suggest temporal variation in the host transcriptional response, reflecting differences in physiological stage, parasite exposure, and immune status across sampling periods. Reactome enrichment was based on sheep‐to‐human ortholog mapping and was therefore interpreted as supportive cross‐species functional evidence. Disease‐labeled KEGG and reactome pathways were considered indicators of shared molecular processes rather than evidence of the corresponding diseases. Given the limited number of DEGs contributing to ORA terms, the enrichment results were interpreted primarily as hypothesis‐generating functional patterns that require validation in independent animals or by targeted molecular assays.

### 4.5. Limitations of the Study

A limitation of this study is that the two sampling points occurred during pregnancy and late lactation. The periparturient period is associated with a relaxation of immunity against GINs [53], particularly around lambing and early lactation, and may influence both parasite burden and host gene expression [54]. Furthermore, the small sample size was a major limitation of the transcriptomic analysis. Group sizes were five low‐FEC and five high‐FEC ewes in February and five low‐FEC and four high‐FEC ewes in July. Statistical power and the expected false‐negative rate were not quantitatively estimated; the assessment of these limitations is therefore qualitative. Limited biological replication may reduce power to detect modest expression differences and increase the risk of false negatives. It may also increase the influence of individual animals on the estimated dispersion and fold changes. Thus, the absence of significant fecal DEGs does not establish equivalent expression between groups, and the blood DEGs require validation in a larger cohort. Another limitation is the seasonal fluctuation of GIN infection pressure within the herd. Group assignment was based on three FEC measurements within one screening season. Although previous annual FEC screening was reported for the flock, this study did not evaluate the repeatability or heritability of the selected phenotypes. Repeated measurements within one season therefore do not establish stable or heritable resistance. Because parasite species composition and individual worm burdens were not determined, the observed associations could not be attributed to a particular GIN species or quantified worm burden. These factors limit attribution of the observed phenotypic and transcriptomic differences specifically to host resistance [55]. Although limitations were inherent to the study population and sampling conditions, the observed differences between low‐FEC and high‐FEC ewes remain informative.

## 5. Conclusion

Blood transcriptomic differences were associated with contrasting FEC phenotypes in naturally exposed Katahdin ewes. *C3AR1*, *ADGRE3*, *ATP5IF1*, *RPS3A*, and *BST2B* were identified as candidate genes requiring validation in larger, independent cohorts. Fecal RNA‐seq demonstrated recovery of ovine transcripts but did not demonstrate an ability to distinguish low‐FEC from high‐FEC animals in this cohort; further studies are needed to establish the biological relevance of the transcriptomic findings and their application to selection for GIN resistance.

## Funding

This study was supported by the USDA‐NIFA, Evans‐Allen Capacity Grants Program, Accession No. 7007775, from the U.S. Department of Agriculture′s National Institute of Food and Agriculture.

## Conflicts of Interest

The authors declare no conflicts of interest.

## Supporting information


**Supporting Information** Additional supporting information can be found online in the Supporting Information section. Table S1 provides gastrointestinal reference annotations for host genes detected in fecal RNA sequencing, including primary and secondary reference compartments and expression values from the sheep atlas. Table S2 summarizes input read pairs, uniquely mapped read pairs, and unique mapping percentages for individual fecal RNA sequencing libraries. Figure S1 presents additional Gene Ontology gene set enrichment results. Figure S2 presents KEGG and Reactome overrepresentation analysis of differentially expressed genes in blood collected in February and July.

## Data Availability

The data that support the findings of this study are available from the corresponding author upon reasonable request.
