# Supplementary material for: Analysis of Blood and Fecal Transcriptomes to Assess the Gastrointestinal Nematode Resistance in Naturally Exposed Katahdin Ewe
Source: J Parasitol Res. 2026 Nov 14;2026:3094932. doi: 10.1155/japr/3094932 (PMC13768998; doi:10.1155/japr/3094932)
Supplement: Supplementary file 1 [file JAPR-2026-3094932-s001.docx]

**Supplementary Table S1.** Complete atlas-based gastrointestinal categorization of 233 fecal host-gene records.

| **Record** | **Gene symbol** | **Primary GI group** | **Secondary GI group** | **Primary mean TPM** | **Secondary mean TPM** | **Overall GI TPM range** |
| --- | --- | --- | --- | --- | --- | --- |
| 1 | ARL4A | Rumen | Abomasum | 121.67 | 12 | 2-316 |
| 2 | B4GALT7 | Rumen | Small Intestine | 104.67 | 14 | 3-291 |
| 3 | FNTA | Rumen | Abomasum | 70.33 | 38 | 10-154 |
| 4 | H2AZ1 | Rumen | Large Intestine | 206.67 | 122.38 | 17-400 |
| 5 | LATS1 | Rumen | Small Intestine | 25.33 | 20 | 4-40 |
| 6 | MTOR | Rumen | Large Intestine | 31.67 | 12.25 | 7-70 |
| 7 | PHKG2 | Rumen | Small Intestine | 143.33 | 20 | 4-397 |
| 8 | RAB28 | Rumen | Small Intestine | 51.33 | 34 | 12-66 |
| 9 | RAB4A | Rumen | Large Intestine | 14.67 | 9.75 | 2-29 |
| 10 | RABL2B | Rumen | Small Intestine | 54.33 | 23 | 5-100 |
| 11 | SS18L2 | Rumen | Large Intestine | 46.67 | 40 | 19-80 |
| 12 | TES | Rumen | Large Intestine | 67.67 | 9.14 | 0-143 |
| 13 | UBB | Rumen | Large Intestine | 2295.33 | 736.12 | 309-5648 |
| 14 | csnk2b | Rumen | Large Intestine | 122.33 | 33 | 14-304 |
| 15 | ndufb8 | Rumen | Large Intestine | 134.33 | 85.38 | 42-270 |
| 16 | ran | Rumen | Large Intestine | 117 | 53.25 | 24-251 |
| 17 | ABCG2 | Small Intestine | Large Intestine | 48 | 29.88 | 2-131 |
| 18 | ABCB1 | Small Intestine | Large Intestine | 18.5 | 9 | 0-37 |
| 19 | ABHD5 | Rumen | Large Intestine | 11.67 | 4.62 | 2-27 |
| 20 | ACACA | Large Intestine | Rumen | 14.25 | 13 | 2-22 |
| 21 | ACTB | Small Intestine | Abomasum | 462 | 442 | 67-1004 |
| 22 | AKIRIN2 | Rumen | Abomasum | 54.33 | 21.33 | 4-119 |
| 23 | ALDH1A1 | Abomasum | Rumen | 123.67 | 114.33 | 1-318 |
| 24 | ANXA2 | Large Intestine | Small Intestine | 246.75 | 229 | 6-957 |
| 25 | AQP1 | Small Intestine | Large Intestine | 94 | 46.5 | 2-152 |
| 26 | ARAF | Small Intestine | Large Intestine | 11 | 9.12 | 4-12 |
| 27 | ARF1 | Rumen | Small Intestine | 165 | 133 | 43-230 |
| 28 | ARF6 | Large Intestine | Small Intestine | 49.12 | 39 | 8-174 |
| 29 | ARFIP2 | Small Intestine | Large Intestine | 28 | 25.88 | 3-70 |
| 30 | ARL1 | Rumen | Small Intestine | 88.33 | 78 | 14-132 |
| 31 | ARL2BP | Rumen | Small Intestine | 43.33 | 32 | 5-90 |
| 32 | ARL4C | Large Intestine | Small Intestine | 9.46 | 7 | 0.7-20 |
| 33 | ARL5A | Small Intestine | Abomasum | 31 | 24.33 | 7-32 |
| 34 | ARNTL | Rumen | Small Intestine | 10.67 | 9 | 1-17 |
| 35 | ATF4 | Large Intestine | Rumen | 60.5 | 58.67 | 32-102 |
| 36 | ATP1A1 | Large Intestine | Rumen | 58.75 | 53 | 14-95 |
| 37 | ATP1B1 | Large Intestine | Abomasum | 132.75 | 113.67 | 4-326 |
| 38 | ATP5PD | Large Intestine | Rumen | 21.25 | 18.33 | 11-40 |
| 39 | ATP6 | Large Intestine | Rumen | 1633.88 | 997 | 302-4241 |
| 40 | ATP6V0C | Rumen | Large Intestine | 6.33 | 5.59 | 0-12 |
| 41 | ATP8 | Large Intestine | Rumen | 885.5 | 736 | 137-2290 |
| 42 | B2M | Small Intestine | Abomasum | 3397 | 2656.67 | 222-6293 |
| 43 | B2M | Small Intestine | Abomasum | 3397 | 2656.67 | 222-6293 |
| 44 | BCO1 | None | None | NA | NA | 0-1 |
| 45 | BHLHE40 | Large Intestine | Small Intestine | 14.5 | 8 | 3-44 |
| 46 | BLOC1S4 | Abomasum | Small Intestine | 5 | 5 | 0.5-9 |
| 47 | BMPR2 | Small Intestine | Large Intestine | 25 | 19.12 | 8-33 |
| 48 | BTG2 | Large Intestine | Small Intestine | 17.5 | 17 | 3-42 |
| 49 | BTG3 | Rumen | Small Intestine | 24 | 20 | 2-52 |
| 50 | CAPN2 | Small Intestine | Large Intestine | 54 | 38.12 | 12-82 |
| 51 | CAST | Rumen | Large Intestine | 47.67 | 39.88 | 9-141 |
| 52 | CCND3 | Small Intestine | Abomasum | 10 | 8.33 | 1-16 |
| 53 | CD247 | Abomasum | Rumen | 34.67 | 15.33 | 1-88 |
| 54 | CD3D | Abomasum | Rumen | 16.9 | 8.7 | 0-49 |
| 55 | CD3E | Abomasum | Small Intestine | 24.27 | 10 | 0-70 |
| 56 | CD52 | Abomasum | Large Intestine | 76.67 | 37.19 | 0-240 |
| 57 | CD53 | Large Intestine | Abomasum | 12.8 | 10 | 0.3-81 |
| 58 | CDK5 | Large Intestine | Rumen | 18.38 | 15.67 | 5-35 |
| 59 | CDKN1A | Rumen | Large Intestine | 25 | 8.72 | 0.8-52 |
| 60 | CIB1 | Rumen | Small Intestine | 34.67 | 18 | 2-73 |
| 61 | CLDN4 | Small Intestine | Large Intestine | 47 | 21.12 | 0-106 |
| 62 | CLDN7 | Small Intestine | Rumen | 70 | 66.67 | 0-186 |
| 63 | COX1 | Large Intestine | Rumen | 10070.5 | 6474 | 2119-28987 |
| 64 | COX2 | Large Intestine | Rumen | 4970.88 | 3519.33 | 1665-11632 |
| 65 | COX3 | Large Intestine | Rumen | 3323.25 | 2024 | 686-9170 |
| 66 | CRY2 | Large Intestine | Rumen | 11.75 | 9.33 | 2-33 |
| 67 | CTSB | Large Intestine | Small Intestine | 172 | 140 | 13-972 |
| 68 | CXCL10 | Small Intestine | Abomasum | 95 | 53 | 3-95 |
| 69 | CXCL8 | Rumen | None | 4.4 | NA | 0-13 |
| 70 | CYB5A | Small Intestine | Abomasum | 71 | 58.67 | 16-176 |
| 71 | CYTB | Large Intestine | Rumen | 1527.75 | 983.67 | 333-4845 |
| 72 | Cxcl1 | None | None | NA | NA | 0-5 |
| 73 | DEGS1 | Small Intestine | Large Intestine | 12 | 11.25 | 1-43 |
| 74 | DGAT1 | Rumen | Large Intestine | 17.67 | 7.12 | 2-23 |
| 75 | DYNLT3 | Small Intestine | Rumen | 59 | 56.33 | 24-85 |
| 76 | EDN1 | Small Intestine | Rumen | 5 | 2.7 | 0-8 |
| 77 | EGR1 | Abomasum | Large Intestine | 5.4 | 3.29 | 0.3-15 |
| 78 | EIF5A | Rumen | Large Intestine | 22 | 20.5 | 8-49 |
| 79 | EPCAM | Rumen | Small Intestine | 188 | 137 | 0.1-303 |
| 80 | ERP29 | Large Intestine | Small Intestine | 79.25 | 79 | 20-231 |
| 81 | FBXL3 | Abomasum | Small Intestine | 32.33 | 30 | 11-46 |
| 82 | FCGR3A | Abomasum | Small Intestine | 84 | 45 | 0.9-239 |
| 83 | FH | Large Intestine | Rumen | 33.88 | 26.33 | 15-54 |
| 84 | FOXO3 | Large Intestine | Small Intestine | 12.12 | 11 | 4-23 |
| 85 | FURIN | Large Intestine | Small Intestine | 20.75 | 16 | 3-90 |
| 86 | GDI2 | Large Intestine | Rumen | 67.62 | 64.67 | 14-151 |
| 87 | GGCX | Small Intestine | Large Intestine | 24 | 10.88 | 2-24 |
| 88 | GSN | Small Intestine | Large Intestine | 224 | 62.88 | 4-224 |
| 89 | GSTA1 | Abomasum | Large Intestine | 105.03 | 51.9 | 0-412 |
| 90 | HAMP | None | None | NA | NA | 0-0.9 |
| 91 | HSD11B1 | Small Intestine | Rumen | 16 | 3.9 | 0.1-16 |
| 92 | HSD11B2 | Rumen | Small Intestine | 9.53 | 2 | 0-28 |
| 93 | HSD17B2 | Rumen | Large Intestine | 14.7 | 1.46 | 0-44 |
| 94 | HTT | Large Intestine | Rumen | 13 | 12.33 | 2-34 |
| 95 | IDH1 | Large Intestine | Rumen | 24.75 | 21.33 | 3-87 |
| 96 | IDO1 | Abomasum | Large Intestine | 4.33 | 2.75 | 2-7 |
| 97 | IGFBP7 | Small Intestine | Abomasum | 191 | 138.33 | 16-266 |
| 98 | IL15 | Small Intestine | Abomasum | 9 | 6.3 | 0.3-15 |
| 99 | IL1A | Small Intestine | Abomasum | 4 | 3 | 0-11 |
| 100 | IL1B | Abomasum | None | 1 | NA | 0-2 |
| 101 | IL2RA | Small Intestine | None | 1 | NA | 0-5 |
| 102 | ILK | Rumen | Small Intestine | 42.33 | 35 | 15-65 |
| 103 | IRAK4 | Small Intestine | Abomasum | 12 | 9 | 2-15 |
| 104 | IRF2 | Small Intestine | Abomasum | 23 | 22 | 5-37 |
| 105 | IRF6 | Small Intestine | Large Intestine | 17 | 11.14 | 0-36 |
| 106 | ITGAL | Abomasum | Small Intestine | 11.33 | 5 | 0.2-30 |
| 107 | ITGAM | Small Intestine | Abomasum | 7 | 6.67 | 0.4-10 |
| 108 | ITGAV | Small Intestine | Abomasum | 34 | 29.67 | 6-83 |
| 109 | ITGB1 | Small Intestine | Abomasum | 133 | 118 | 17-200 |
| 110 | ITGB2 | Abomasum | Small Intestine | 18 | 10 | 0.3-48 |
| 111 | ITGB6 | Small Intestine | Rumen | 13 | 7.03 | 0-17 |
| 112 | ITLN2 | None | None | NA | NA | 0-1 |
| 113 | ITM2B | Small Intestine | Abomasum | 244 | 239.33 | 52-351 |
| 114 | ITM2C | Small Intestine | Abomasum | 61 | 50.67 | 3-74 |
| 115 | KLF4 | Rumen | Small Intestine | 28.77 | 6 | 0.3-56 |
| 116 | KLK1 | Rumen | None | 5.33 | NA | 0-14 |
| 117 | LAMC2 | Rumen | Large Intestine | 4.03 | 1.04 | 0-8 |
| 118 | LCK | Abomasum | Large Intestine | 11.07 | 4.33 | 0.2-31 |
| 119 | LIPA | Abomasum | Small Intestine | 89.33 | 79 | 3-238 |
| 120 | LIPE | Rumen | Abomasum | 10.17 | 2.53 | 0.4-29 |
| 121 | LYN | Abomasum | Small Intestine | 17 | 15 | 0.5-46 |
| 122 | LYZ | Abomasum | Rumen | 51.33 | 13.1 | 0.3-150 |
| 123 | MAPK13 | Small Intestine | Rumen | 26 | 21 | 0.3-55 |
| 124 | MAPK14 | Small Intestine | Large Intestine | 35 | 34.38 | 8-72 |
| 125 | MAPKAP1 | Rumen | Small Intestine | 26.67 | 19 | 5-36 |
| 126 | MET | Small Intestine | Large Intestine | 27 | 11.88 | 1-37 |
| 127 | MMP13 | None | None | NA | NA | 0-0.4 |
| 128 | MRC1 | Abomasum | Small Intestine | 38.63 | 27 | 0.3-111 |
| 129 | MRCL3 | Small Intestine | Abomasum | 298 | 215 | 44-495 |
| 130 | MX1 | Small Intestine | Large Intestine | 101 | 19.5 | 2-101 |
| 131 | MX2 | Small Intestine | Large Intestine | 26 | 5.29 | 0.4-26 |
| 132 | MYC | Abomasum | Small Intestine | 9.63 | 9 | 0.2-20 |
| 133 | MYD88 | Small Intestine | Large Intestine | 12 | 6.97 | 0.8-14 |
| 134 | NCK1 | Rumen | Abomasum | 27.67 | 19 | 4-41 |
| 135 | ND1 | Large Intestine | Rumen | 926.88 | 594.67 | 220-1969 |
| 136 | ND2 | Large Intestine | Rumen | 1017.62 | 606.33 | 209-2729 |
| 137 | ND3 | Large Intestine | Rumen | 1361.62 | 1034.33 | 327-2982 |
| 138 | ND4 | Large Intestine | Rumen | 1337.12 | 904.67 | 280-3869 |
| 139 | ND4L | Large Intestine | Rumen | 4.62 | 3.2 | 0-13 |
| 140 | ND5 | Large Intestine | Rumen | 424 | 295.67 | 88-1137 |
| 141 | ND6 | Large Intestine | Rumen | 88.62 | 45 | 17-199 |
| 142 | NDUFB6 | Large Intestine | Rumen | 15.38 | 12.33 | 7-25 |
| 143 | NFKBIA | Small Intestine | Abomasum | 16 | 12.33 | 3-25 |
| 144 | NR3C1 | Abomasum | Large Intestine | 19.33 | 13.25 | 8-28 |
| 145 | NR5A2 | Abomasum | Small Intestine | 6.97 | 3 | 0-20 |
| 146 | PDIA3 | Small Intestine | Rumen | 283 | 237.67 | 30-456 |
| 147 | PDXK | Small Intestine | Rumen | 59 | 28.67 | 2-59 |
| 148 | PGD | Small Intestine | Rumen | 45 | 44.67 | 8-79 |
| 149 | PIM1 | Rumen | Abomasum | 44.67 | 11.33 | 1-121 |
| 150 | PON2 | Large Intestine | Small Intestine | 42.12 | 28 | 4-134 |
| 151 | PPARG | Abomasum | Large Intestine | 18.4 | 16.01 | 0.2-109 |
| 152 | PRKAR1A | Rumen | Large Intestine | 60 | 37.38 | 15-107 |
| 153 | PSMB8 | Small Intestine | Abomasum | 8 | 6.93 | 0-19 |
| 154 | PTGS2 | Small Intestine | Large Intestine | 32 | 6.53 | 0.1-32 |
| 155 | PYGB | Large Intestine | Rumen | 24.5 | 23.33 | 4-53 |
| 156 | PYGL | Rumen | Abomasum | 24 | 22 | 4-47 |
| 157 | RAB1A | Large Intestine | Small Intestine | 120.5 | 108 | 50-272 |
| 158 | RAB21 | Large Intestine | Abomasum | 8.5 | 8.33 | 4-19 |
| 159 | RAB5A | Rumen | Abomasum | 30.33 | 28.67 | 6-40 |
| 160 | RAC1 | Large Intestine | Small Intestine | 61.12 | 55 | 16-161 |
| 161 | RACK1 | Abomasum | Rumen | 168.33 | 167.33 | 38-268 |
| 162 | RHEB | Abomasum | Small Intestine | 45.33 | 45 | 26-65 |
| 163 | RHOA | Rumen | Small Intestine | 83.33 | 66 | 21-135 |
| 164 | RHOB | Abomasum | Small Intestine | 36 | 29 | 6-66 |
| 165 | RHOF | Abomasum | Large Intestine | 5.33 | 4.12 | 0.5-12 |
| 166 | RHOH | Abomasum | Large Intestine | 20.03 | 7.91 | 0-59 |
| 167 | RPA1 | Rumen | Small Intestine | 25.67 | 24 | 10-33 |
| 168 | RSU1 | Small Intestine | Abomasum | 36 | 32.33 | 4-46 |
| 169 | SDR16C5 | Large Intestine | Rumen | 9.21 | 4.67 | 0.3-52 |
| 170 | SERPINA1 | Large Intestine | Abomasum | 72.9 | 1.7 | 0-565 |
| 171 | SERPINB1 | Abomasum | Small Intestine | 16.67 | 12 | 0.6-41 |
| 172 | SERPINE1 | Small Intestine | Abomasum | 3 | 1.7 | 0-3 |
| 173 | SFXN1 | Large Intestine | Small Intestine | 22.5 | 21 | 2-57 |
| 174 | SKP1 | Rumen | Large Intestine | 141.33 | 132.5 | 53-265 |
| 175 | SLC17A5 | Large Intestine | Rumen | 18.38 | 15.67 | 3-72 |
| 176 | SLC25A11 | Large Intestine | Small Intestine | 35.38 | 26 | 6-62 |
| 177 | SLC25A15 | Large Intestine | Rumen | 2.56 | 1.93 | 0.5-6 |
| 178 | SLC25A33 | Rumen | Abomasum | 2.67 | 1.67 | 0.4-6 |
| 179 | SLC25A38 | Rumen | Small Intestine | 14.67 | 10 | 3-30 |
| 180 | SLC26A2 | Abomasum | Rumen | 30 | 27.33 | 0.5-83 |
| 181 | SLC2A3 | Rumen | Large Intestine | 42.67 | 25.5 | 1-133 |
| 182 | SLC30A7 | Large Intestine | Abomasum | 32.12 | 25.67 | 5-62 |
| 183 | SLC39A7 | Rumen | Large Intestine | 18 | 17.75 | 2-37 |
| 184 | SLC5A1 | Rumen | Large Intestine | 7.67 | 6.89 | 0-50 |
| 185 | SLC66A2 | Rumen | Small Intestine | 7.33 | 7 | 2-12 |
| 186 | SLCO2A1 | Small Intestine | Large Intestine | 27 | 12.66 | 0.1-66 |
| 187 | SMAD4 | Small Intestine | Abomasum | 45 | 43 | 13-60 |
| 188 | SMPD1 | Abomasum | Small Intestine | 10.33 | 10 | 3-20 |
| 189 | SNRPA1 | Rumen | Small Intestine | 19.67 | 19 | 9-34 |
| 190 | SQLE | Large Intestine | Abomasum | 21.49 | 20.33 | 0.9-72 |
| 191 | SRI | Rumen | Abomasum | 82.33 | 36.33 | 3-197 |
| 192 | SRSF3 | Small Intestine | Rumen | 196 | 192.33 | 52-275 |
| 193 | ST3GAL4 | Abomasum | Large Intestine | 32.67 | 17.5 | 3-76 |
| 194 | STAR | Rumen | None | 1.57 | NA | 0-4 |
| 195 | STAT1 | Rumen | Small Intestine | 33.33 | 21 | 5-70 |
| 196 | TAPBP | Small Intestine | Rumen | 21 | 17 | 3-25 |
| 197 | THRB | Rumen | Abomasum | 10.67 | 6.33 | 0.1-21 |
| 198 | TIMP1 | Small Intestine | Abomasum | 32 | 17.33 | 3-32 |
| 199 | TIMP2 | Small Intestine | Rumen | 181 | 142 | 6-349 |
| 200 | TIMP3 | Small Intestine | Abomasum | 124 | 87 | 15-131 |
| 201 | TLR2 | Small Intestine | Abomasum | 16 | 6.67 | 0.4-17 |
| 202 | TLR4 | Small Intestine | Abomasum | 12 | 9.33 | 0.5-23 |
| 203 | TLR6 | Rumen | Abomasum | 5 | 5 | 0.7-8 |
| 204 | TMEM154 | Large Intestine | Abomasum | 5.9 | 4.8 | 0-23 |
| 205 | TNF | Abomasum | None | 5.23 | NA | 0-12 |
| 206 | TPM1 | Large Intestine | Abomasum | 248 | 155.33 | 16-1603 |
| 207 | TRA2A | Rumen | Small Intestine | 56 | 46 | 10-70 |
| 208 | TRPM7 | Small Intestine | Large Intestine | 24 | 18.5 | 9-24 |
| 209 | TSPO | Small Intestine | Rumen | 30 | 23.67 | 2-64 |
| 210 | TUBA4A | Large Intestine | Abomasum | 70.88 | 26.33 | 3-137 |
| 211 | TXN | Large Intestine | Small Intestine | 177.88 | 170 | 32-584 |
| 212 | UCP2 | Rumen | Abomasum | 19 | 12.33 | 0.9-36 |
| 213 | VEGFA | Large Intestine | Abomasum | 18.12 | 14 | 2-37 |
| 214 | YWHAZ | Rumen | Large Intestine | 175.67 | 162.75 | 22-288 |
| 215 | ZDHHC7 | Rumen | Large Intestine | 12.33 | 11.38 | 6-20 |
| 216 | ZFAND5 | Abomasum | Small Intestine | 29.33 | 29 | 11-48 |
| 217 | ZFP36 | Abomasum | Small Intestine | 4.33 | 4 | 0.4-7 |
| 218 | bckdha | Rumen | Large Intestine | 18.33 | 11.38 | 4-38 |
| 219 | isg15 | Small Intestine | Large Intestine | 75 | 13.44 | 0-75 |
| 220 | pgk1 | Large Intestine | Abomasum | 70.25 | 57 | 30-110 |
| 221 | rpl23a | Small Intestine | Abomasum | 1731 | 1365 | 272-2089 |
| 222 | rpl23a | Small Intestine | Abomasum | 1731 | 1365 | 272-2089 |
| 223 | rpsa | Rumen | Small Intestine | 506.67 | 506 | 147-830 |
| 224 | slc25a10 | Rumen | Small Intestine | 8 | 6 | 0.8-16 |
| 225 | uba52 | Rumen | Small Intestine | 568.67 | 385 | 136-1061 |
| 226 | vdac1 | Large Intestine | Rumen | 35.25 | 31 | 8-59 |

**Supplementary Figure S1.** Additional GO GSEA results. (a) February GO molecular function, (b) July GO molecular function, and (c) July GO cellular component. Genes were ranked by the signed DESeq2 Wald statistic; point size indicates the number of core-enrichment genes, GeneRatio indicates their proportion within the corresponding gene set, and color denotes the Benjamini–Hochberg-adjusted P value. Panels a and b include categories that did not all meet the adjusted P ≤ 0.05 threshold; all displayed categories in panel c met the threshold. No February GO cellular-component category met adjusted P ≤ 0.05.

**Supplementary Figure S2.** Over-representation analysis of differentially expressed genes. (a) February KEGG, (b) February Reactome using human orthologs, (c) July KEGG, and (d) July Reactome using human orthologs. Point size indicates the number of overlapping DEGs, GeneRatio indicates their proportion within the corresponding gene set, and color denotes the Benjamini–Hochberg-adjusted P value. ORA used the prespecified DEG threshold; no July Reactome term met adjusted P ≤ 0.05.

**Supplementary Table S2.** Sample-level recovery of ovine transcripts from fecal RNA-seq libraries.

| **Library** | **Ewe ID in source file** | **Input read pairs** | **Uniquely mapped read pairs** | **Unique mapping (%)** |
| --- | --- | --- | --- | --- |
| S19 | 1928 | 62,749,978 | 358,915 | 0.57 |
| S20 | 2016 | 54,321,447 | 269,417 | 0.50 |
| S21 | 2020 | 56,433,683 | 403,640 | 0.72 |
| S22 | 2171 | 54,302,182 | 451,118 | 0.83 |
| S23 | 2278 | 60,521,842 | 215,662 | 0.36 |
| S24 | 2286 | 58,590,576 | 498,515 | 0.85 |
| S25 | 3018 | 55,860,203 | 498,187 | 0.89 |
| S26 | 3040 | 65,242,246 | 477,403 | 0.73 |
| S27 | 3044 | 56,518,390 | 3,025,723 | 5.35 |
| S28 | 3045 | 51,439,376 | 166,395 | 0.32 |
